# Early change and new onset of vascular pathology in cervical artery dissection: implications for surveillance

**DOI:** 10.1093/esj/aakag117

**Published:** 2026-10-05

**Authors:** Robin Seihu, Tobias Hofmann, Frank Dömges, Hakan Cangür, Martin Döltz, Jens Neumann, Steffen Eue, Alexander Knörnschild, Daniel Behme, Stefanie Schreiber, Michael Görtler, for the TASC-SAN Network

**Affiliations:** Department of Neurology, Magdeburg University Hospital, Magdeburg, Germany; Central Emergency Department, Magdeburg University Hospital, Magdeburg, Germany; Department of Neurology, Harzklinikum Wernigerode, Wernigerode, Germany; Department of Neurology, Klinikum Wolfsburg, Wolfsburg, Germany; Department of Neurology, Ameos-Klinikum Haldensleben, Haldensleben, Germany; Department of Neurology, MEDIAN-Klinikum Flechtingen, Flechtingen, Germany; Department of Neurology, Magdeburg University Hospital, Magdeburg, Germany; Department of Neurology, Ameos-Klinikum Haldensleben, Haldensleben, Germany; Department of Neurology, Altmark-Klinikum Gardelegen, Gardelegen, Germany; Department of Neurology, Ameos-Klinikum Bernburg, Bernburg, Germany; Department of Neurology, Ameos-Klinikum Bernburg, Bernburg, Germany; Department of Neuroradiology, Magdeburg University Hospital, Magdeburg, Germany; Department of Neurology, Magdeburg University Hospital, Magdeburg, Germany; Department of Neurology, Magdeburg University Hospital, Magdeburg, Germany

**Keywords:** anticoagulants, antiplatelet agents, cervical artery dissection, dissection recurrence, recanalisation

## Abstract

**Introduction:**

Antithrombotic therapy is central to stroke prevention after spontaneous cervical artery dissection (sCAD), but its efficacy may depend on the underlying vascular morphology. We examined the temporal course of vascular changes after sCAD.

**Patients and methods:**

Consecutive patients with first-time sCAD underwent prospective serial duplex sonography for up to 10 years. In this retrospective cohort analysis, pathology change was defined as recanalisation of an occluded artery or progression from stenosis to occlusion. New pathology resulting from dissection recurrence was considered if an occlusion occurred in the presence of pre-existing stenosis or vice versa.

**Results:**

In total, 159 patients (83 men; mean age 44.8 years) with first-time sCAD were followed-up starting 1.3 days (median) after symptom onset. Bilateral dissections occurred in 6 patients and recurrent dissections in 26 arteries, resulting in follow-up of 191 arteries (108 internal carotid, 83 vertebral). Recanalisation occurred in 40/80 occluded arteries (cumulative rate 70.4%), whereas progression to occlusion occurred in 9/109 stenosed arteries (10.0%). All changes except one (recanalisation) occurred within 4 months. Recurrent dissection occurred within 6 weeks in 20 patients (14.7%) and between 2 and 7 years in 7 patients (13.7%). Overall, 62 vascular changes or new pathologies were observed in 50 patients (31.4%), with 42 occurring within the first 6 weeks.

**Conclusions:**

Under intensive surveillance, vessel morphology after sCAD is highly dynamic during the early phase, with frequent recanalisation and recurrent dissection. Structured early vascular follow-up may be important if evolving vessel morphology is to guide future individualised antithrombotic treatment strategies.

## Graphical Abstract

**Graphical Abstract:**
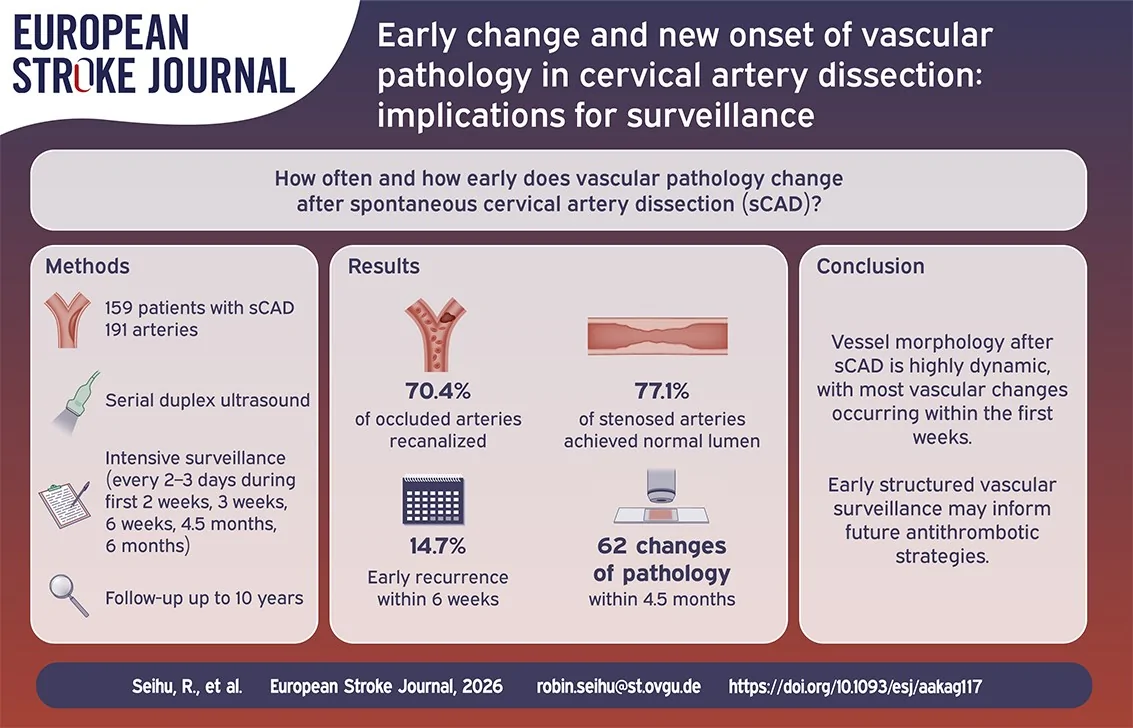

## Introduction

Spontaneous cervical artery dissection (sCAD) accounts for only up to 2% of all ischaemic strokes, but is the leading stroke aetiology of up to 25% in young adults with an annual incidence rate of 2.6–2.9 per 100,000.^1–4^ The predominant finding of sCAD is an intramural haematoma caused by a ruptured vasa vasorum in the medial/adventitial border or a tear in the tunica intima of the vessel wall with subsequent dissecting haematoma.^5^^,^^6^ Steno-occlusion of the vessel lumen and compression of surrounding tissue and nerves may result in cerebral ischaemia and loss of function in adjacent peripheral nerves, respectively.^6^^,^^7^

Antithrombotic agents, ie, antiplatelet agents and anticoagulants, are recommended for therapy and prevention of subsequent cerebral ischaemia, though no standard treatment has been established yet.^8^^,^^9^ A recent observational study suggests that efficacy of antiplatelet agents and anticoagulants may vary according to the underlying vascular pathology, ie, stenosis or occlusion.^10^ If this were confirmed, it would be necessary to adjust the antithrombotic medication to the respective vascular finding. However, occlusion and stenosis are not stationary conditions after sCAD. In observational studies, approximately 60% of dissection-related occlusions have been shown to recanalise spontaneously (at least to stenosis) after 6–9 months, and initial stenoses have been shown to progress to occlusion within this time.^11–14^ Recurrent dissection within this period with subsequent occlusion or stenosis is another cause of a changing vascular pathology in a patient. Its frequency is reported very inconsistently in the literature, ranging from 0.9% to 26.3% within the first month after a preceding dissection.^12^^,^^15^

Knowledge of the early temporal dynamics of dissection-related vessel pathology change after an sCAD and its timely detection might be of therapeutic relevance in the future. Both have been addressed in only a few studies and not comprehensively. This study investigates the early recanalisation and progression of dissection-related occlusion and stenosis, respectively, and their new occurrence as a result of an early recurrent dissection. We aimed to determine the timing and frequency of changes in vascular pathology following spontaneous cervical artery dissection, including recanalisation, progression to occlusion and recurrent dissection, and to explore their potential therapeutic relevance in patients with sCAD.

## Patients and methods

### Patients’ selection and follow-up

Patients referred to our stroke centre, directly or via a satellite hospital of the Telemedical Acute Stroke Care Sachsen-Anhalt (TASC-SAN) network, who presented with symptoms or had undergone vascular diagnostics (CTA, MRA, colour-coded duplex sonography) suggesting spontaneous cervical artery dissection underwent subsequent MRI to verify the dissecting pathology. The vascular course of a dissected artery was followed prospectively by repetitive duplex sonographic examinations every 2–3 days within the first 2 weeks, after 3 weeks, 6 weeks, 4.5 months and thereafter every 6 months. After the introduction of duplex sonography as an immediate initial examination, the observation of very early vascular changes led us to establish this standardised follow-up protocol for patients with stroke and cerebral macroangiopathy, including cervical artery dissection. Follow-up was terminated, if the patient or his/her outpatient physician refused further examinations at our centre. If a patient failed to attend a scheduled appointment, we contacted him/her by telephone and asked for the reason of incompliance.

For the present evaluation, we selected all consecutive patients with the diagnosis of a first-time spontaneous cervical artery dissection who were treated at our institution between 1998 and 2024. Follow-up evaluation was censored when a patient had undergone endovascular therapy or follow-up had been terminated.

All examinations were performed within standard clinical practice. Data were collected, entered into our institution’s stroke registry and evaluated in accordance with federal and national data protection laws so that the responsible ethics committee of the Otto-von-Guericke University waived the need for patient’s consent (214/2025).

### Vascular diagnostic

Duplex sonography included carotid artery examination from the clavicle to the extracranial skull base, examination of the vertebral artery from its origin to the distal limb of the atlas loop, as well as transcranial and transnuchal examination of the basal intracranial arteries. Diagnosis of spontaneous internal carotid artery (ICA) dissection was based on the presence of an extracranial vessel pathology distal to the bifurcation level compatible with arterial dissection, ie, stenosis or occlusion with a causative wall haematoma or 2 lumina with different flow characteristics separated by an (intimal) flap.^16^^,^^17^ Stenoses could be visualised directly using CTA, contrast-enhanced MRA or colour-coded duplex sonography. They were diagnosed in the presence of a segmental reduction in lumen diameter compared to the unaffected arterial segment before the stenosis. Due to the reduction in blood flow volume, the correlation between intrastenotic peak systolic velocity (PSV) and the degree of stenosis—which is well-known from atherosclerotic stenoses—does not apply to dissection-related stenoses; therefore, we did not perform any further quantification of the degree of stenosis, eg, based on PSV. The normalisation of the previously reduced blood flow volume, as measured in the proximal internal carotid artery and resulting from long-segment dissection-induced stenosis near the skull base, also allowed duplex sonography to assume the resolution of the stenosis, with subsequent confirmation by MRI. In the case of an occlusion, duplex sonography revealed a pre-occlusive stump signal in otherwise normal proximal ICA. The corresponding CTA finding was often a fading contrast enhancement at the origin of the artery, primarily in its dorsal half when the patient was lying on their back. This is caused by a pulsating column of contrast agent being denser than blood (Figure 1). Duplex sonography also allowed visualisation of the wall haematoma, distinguished from a thrombus by the increased external vessel diameter.^18^^,^^19^ Otherwise, additional MRI with fat-suppressed T1 weighting at the level of the stenotic pathology confirmed the haematoma and the diagnosis.^20^ Vertebral artery dissection was diagnosed in a comparable way. Non-stenosing and non-occluding dissection pathologies, eg, intimal flaps, were detected by means of angiographic techniques or sonography. In order to distinguish between a methodological and a vascular cause of discrepant findings in duplex sonography and subsequent non-ultrasound modalities, we repeated the ultrasound examination immediately after the non-ultrasound modality during the acute inpatient phase. In patients treated with intravenous thrombolysis or endovascular therapy, the initial vascular pathology (stenosis or occlusion) was determined from vascular imaging performed before treatment.

**Figure 1:**
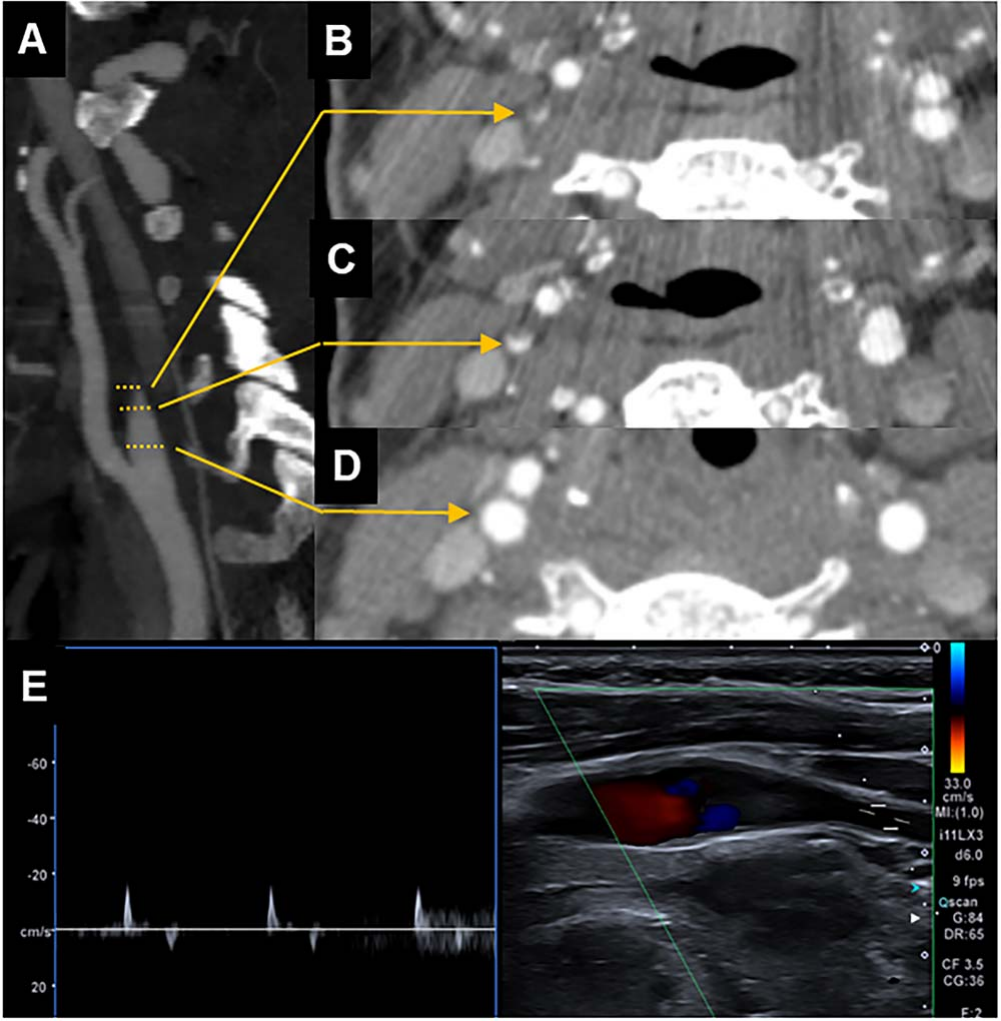
Occluding dissection of the ICA at the extracranial skull base (not shown). Computer tomography angiography with sagittal reconstruction (A) and transverse sections (B–D) of extracranial carotid bifurcation. Fading contrast enhancement in the dorsal half of ICA at its origin. Colour-coded duplex sonography with corresponding pre-occlusive stump signal (E) in otherwise normal proximal ICA. Abbreviation: ICA = internal carotid artery.

### Patients’ clinical workup and treatment

Patients referred with a suspected cerebral ischaemic event underwent standardised clinical work-up which included admission brain CT, follow-up CT and/or MRI, recurrent assessment of neurological and functional deficit, cardiac (eg, Holter monitoring, echocardiography) and laboratory examinations. Standard acute stroke therapy included intravenous thrombolysis, endovascular thrombectomy in case of intracranial large artery occlusion, early administration of antiplatelet agents and stroke unit care. Secondary prevention was carried out according to current guidelines and included antiplatelet agents and anticoagulants. The neurological and the functional deficit were quantified applying the NIHSS and the mRS, respectively. For the present evaluation assessments at admission (NIHSS, mRS), hospital discharge (NIHSS) and 4.5 months after the index event (mRS) were used.

### Outcome

Both, vessel recanalisation and recurrent dissection served as outcome parameters. Time to recanalisation of an occluded artery was determined when follow-up duplex sonography detected a stenosis or even a vessel that had returned to normal from prior occlusion. Time to normalisation was determined when follow-up duplex sonography demonstrated neither former occlusion nor stenosis for the first time. Time to a recurrent dissection was determined when we detected corresponding findings (in asymptomatic patients) at a follow-up examination or when a patient had suffered symptoms diagnosed in subsequent diagnostics as caused by (recurrent) dissection.

To estimate the clinical relevance of both outcome parameters, we used the change of the neurological deficit from admission to discharge—considered as improvement or deterioration if the NIHSS score had decreased or increased by 4 points or more, respectively, and the change of the functional deficit between admission and the 4.5-month follow-up, expressed as difference of the mRS.

### Statistics

For statistical analyses, we used the SPSS software package. Time course of arterial recanalisation and dissecting recurrence was analysed using Kaplan–Meier curve and Cox proportional hazards regression. Variables investigated for interaction with both were patients’ demographics (age and sex), the artery involved (carotid and vertebral artery), non-invasive therapeutic measures (intravenous thrombolysis, antithrombotic prevention) and year of initial dissection. We conducted binary logistic regression analysis and multiple linear regression analysis for paired samples to determine the impact of spontaneous arterial recanalisation and of dissecting recurrence on patients’ neurological and functional outcome, respectively. Values of both variables were included in clinical outcome analyses as they had occurred at discharge and at 4.5 months, respectively. Patients with premature termination of follow-up examinations, eg, in case of endovascular intervention, were excluded from the respective analysis of the clinical outcome. Also excluded were arteries with an initial stenosis or without known vessel pathology at the time of the clinical outcome assessment. Confidence interval (CI) was set at 95% and significance at a *P* value of less than .05 for 2-sided tests.

Finally, we composed times and frequencies of all changes in vascular pathology and marked those, which might result in a switch of antithrombotic prevention from anticoagulants to antithrombotic agents or vice versa: (1) recanalisation of an occluded artery to stenosis/normal, (2) progression of a stenosed artery to occlusion, (3) new onset of an occlusion in case of pre-existing stenosis and (4) vice versa.

## Results

Between 02/1998 and 07/2024, 159 consecutive patients suffering from a first-time cervical artery dissection were followed up. Vascular admission diagnostics performed 1.3 days (median; IQR, 0.3–3.8) after symptoms onset detected single artery dissection in 153 and multiple artery dissection in 6 patients (3 with bilateral ACI and 3 with bilateral VA involvement). Interaction of clinical variables with changes in arterial pathology and recurrence of dissection was analysed in these 159 patients. Mean follow-up period was 3.9 years after excluding those patients with acute endovascular recanalisation (20) or other interventions not enabling close follow-up (4). The demographics, baseline characteristics and clinical findings of these patients are presented in Table 1.

**Table 1:** Demographics, baseline characteristics and clinical findings in 159 consecutive patients admitted with first-time spontaneous cervical artery dissection.

	*n* (%)
**Age, years, mean (SD)**	44.8 (11.7)
**Sex, male**	83 (52.2)
**Hypertension**	83 (52.2)
**Diabetes**	15 (9.4)
**Hypercholesterolaemia**	66 (41.5)
**Body mass index, kg/m^2^, mean (SD)**	25.7 (3.9)
**Migraine**	11 (6.9)
**Fibromuscular dysplasia**	5 (3.1)
**Admission symptoms**	
** Cerebrovascular**	132 (83.0)
** TIA**	37 (23.3)
** Stroke**	95 (59.7)
** Others**	27 (17.0)
**Admission deficit**	
** NIHSS score, Md (IQR)**	1 (0–7)
** mRS, Md (IQR)**	3 (0–4)
**Time from symptoms onset to admission, days, Md (IQR)**	1.3 (0.3-3.8)
**Non-ischaemic symptoms**	
** Acute onset head/neck/cervical pain**	58 (36.5)
** Horner’s syndrome**	14 (8.8)
** Peripheral nerve compression syndrome**	2 (1.3)
** Retrobulbar pressure**	2 (1.3)
** Acute onset pulse synchronous tinnitus**	2 (1.3)
**Symptomatic artery**	
** Internal carotid**	96 (60.4)
** Vertebral**	63 (39.6)
**Vessel pathology of dissected symptomatic artery**	
** Occlusion**	77 (48.4)
** Stenosis**	81 (50.9)
** Other**	1 (0.8)
**Potential trigger**	
** None**	129 (81.1)
** Valsalva-associated manoeuvre**	7 (4.4)
** Chiropractic/massage treatment**	6 (3.8)
** Cervical distortion**	9 (5.7)
** Fall**	8 (5.0)
**Time from trigger to symptoms onset, days, Md (IQR)**	0.6 (0.0–3.0)
**Antithrombotic premedication**	
** None**	152 (95.6)
** Antiplatelet agent**	3 (1.9)
** Anticoagulant**	4 (2.5)

Abbreviations: Md=median; TIA=transient ischaemic attack

During patients’ follow-up observation, 26 additional cervical arteries dissected spontaneously. Thus, the course of arterial pathology (recanalisation, normalisation, progression to occlusion) after a spontaneous cervical artery dissection could be followed-up in 191 arteries. The vascular findings of the 191 dissected arteries are reported in Table 2.

**Table 2:** Vascular findings in 191 spontaneously dissected cervical arteries (108 internal carotid arteries, 83 vertebral arteries).

	All arteries (191)	ICA (108)	VA (83)
	*n* (%)	*n* (%)	*n* (%)
**Side of dissection**			
** Right**	83 (43.5)	48 (44.4)	35 (42.2)
** Left**	108 (56.5)	60 (55.6)	48 (57.8)
**Dissecting vessel pathology**			
** Occlusion**	80 (41.9)	49 (45.4)	31 (37.3)
** Stenosis**	109 (57.1)	57 (52.8)	52 (62.7)
** Intimal flap**	2 (1.0)	2 (1.9)	0
**Involved ICA segments**			
** Isolated proximal petrous**		1 (0.9)	
** Distal extracranial^a^**		107 (99.1)	
**Involved VA segments**			
** Proximal V4 (proximal PICA origin)**			25 (30.1)
** Distal V3 (distal atlas loop)**			48 (57.8)
** Proximal V3 (proximal atlas loop)**			41 (49.4)
** Distal V2 (between first and second vertebra)**			24 (28.9)
** Proximal V2 (between second and sixth vertebra)**			27 (32.5)
** V0 and V1 (proximal 6th vertebra)**			13 (15.6)

Abbreviations: ICA=internal carotid artery; PICA=posterior inferior cerebellar artery; VA=vertebral artery.

Percentage indicates relative frequency of a vascular finding within the cohort of each column.

a With and without proximal petrous segment.

### Spontaneous recanalisation and delayed occlusion

Forty of the 80 occluded arteries recanalised spontaneously: 33 initially to stenosis with secondary normalisation in 17 cases, 7 arteries already initially to normal. Recanalisation occurred in 39 arteries within 4 months, 1 artery recanalised after 9.5 months (Figure 2). Internal carotid artery involvement (as opposed to vertebral artery) was associated with earlier recanalisation (log-rank test; chi-square statistic, 7.625; degree of freedom, 1; *P* = .006) (Figure S1). No association was found for intravenous thrombolysis and for the type of antithrombotic prevention started after the dissection and continued during follow-up (Table S1).

**Figure 2:**
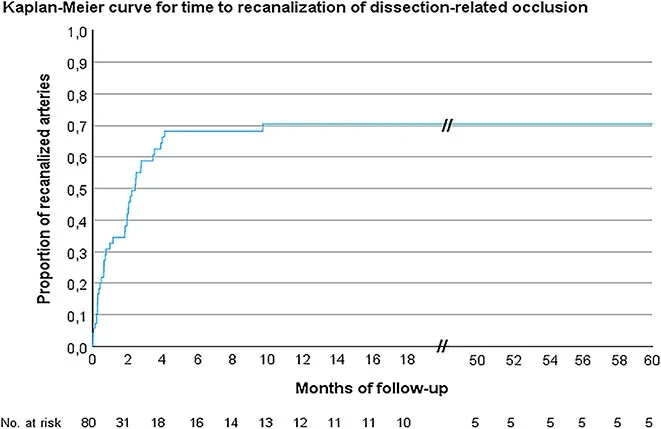
Kaplan–Meier curve for time to spontaneous recanalisation of acute dissection-related cervical artery occlusion in 80 arteries.

Eighty-four of 109 initially stenosed arteries normalised completely. Nine stenoses initially progressed to occlusion within 2 months, of which 5 arteries recanalised to normal via renewed stenosis. A total of 86 stenosed arteries normalised within 1 year, 3 after 18 (2) and 30 months. Initial stenosis (as opposed to occlusion) was associated with earlier normalisation (log-rank test, chi-square statistic, 38.556; degree of freedom, 1; *P* < .001) (Figure S2).

Recanalisation and normalisation did not differ between an initial dissection as compared to a recurrent dissection at follow-up. Figure S3 provides an overview of the changes in vascular pathology resulting from recanalisation, delayed occlusion and normalisation, as observed in the 191 dissected arteries.

In the 159 patients, NIHSS score at hospital discharge had improved in 4 of 13 patients (30.8%) with and in 3 of 41 (7.3%) without spontaneous recanalisation (OR, 5.630; CI, 1.066–29.726; *P* = .042). Spontaneous recanalisation had no effect on the course of the functional deficit. Patients with and without recanalisation had improved from admission to the 4.5-month follow-up (median difference of mRS, −1; IQR, −2 to 0) without additional effect of recanalisation (mean difference of improvement between recanalised/non-recanalised patients, −0.029; CI, −0.832 to 0.773; *P* = .942) (Table S1).

### Dissecting recurrence

Within 6 weeks after their first cervical artery dissection, 20 of the 159 patients suffered a recurrent dissection (7 occlusions, 13 stenoses) (Figure 3). In 17 patients, this early recurrence occurred in the corresponding contralateral artery, 1 patient suffered vertebral artery dissection after initial internal carotid artery involvement and 1 vice versa. In one additional patient, vertebral artery dissected bilaterally after initial carotid artery dissection. Early dissection recurrence was not associated with age, sex, initially involved artery (carotid vs vertebral artery), intravenous thrombolysis, chosen antithrombotic prevention (antiplatelet agents vs anticoagulants) and year of initial dissection. In 14 patients, dissecting recurrence was associated with cerebrovascular ischaemia (TIA in 5, minor stroke in 5, moderate in 3, severe in 1). None of the above mentioned variables served as a predictor for associated ischaemia.

**Figure 3:**
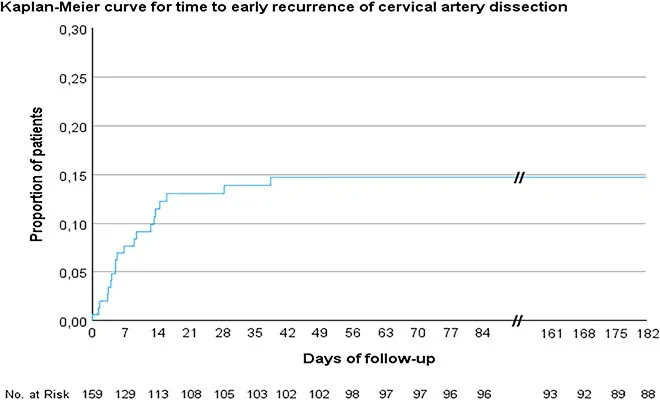
Kaplan–Meier curve for time to early recurrence of cervical artery dissection in 159 patients.

At long-term follow-up, late dissecting recurrence appeared in 7 of the 159 patients between almost 2 and 7 years after the initial dissection (3 occlusions, 4 stenoses), resulting in a cumulative risk of 13.7%. Two of those patients had already experienced early recurrence. Late recurrent dissection was detected in corresponding contralateral arteries (2 patients), internal carotid after initial vertebral artery dissection (2) and vice versa (1) and vertebral artery V3 segment after initial V2-segment involvement (2, both confirmed by fat-suppressed MRI T1 sequences) and was associated with a TIA in 1 patient. Younger age (per year, OR, 0.849; CI, 0.756–0.954; *P* = .001) was associated with late recurrence, but not sex, involved artery, antithrombotic prevention and year of initial dissection. Figure S4 presents time to the first recurrent dissection after initial spontaneous dissection.

Of 191 arteries with a first-time dissection, only 2 vertebral arteries dissected for a second time, both involving V3 segments, in contrast to initially dissected V2 segments. Both occurred late—699 and 732 days after the primary dissection, demonstrated stenoses after normalised preceding stenoses, and were asymptomatic.

In the 159 patients, NIHSS score at hospital discharge had deteriorated in 1 of 18 patients (5.6%) with and in 4 of 104 (3.8%) without recurrent dissection (OR, 1.471; CI, 0.155–13.964; *P* = .556). Patients with dissection recurrence had not improved from admission to the 4.5-month follow-up (median difference of mRS, 0; IQR, −1 to 0) whereas those without recurrence had (median difference of mRS, −1; IQR, −2 to 0). However, the effect of dissection recurrence on the course of the functional deficit was not significant (mean difference of mRS change between recurrence/non-recurrence patients, 0.186; CI, −0.432 to 0.805; *P* = .552).

### Change of dissecting vessel pathology

In 50 of the 159 patients (31.4%), we observed 62 changes of arterial vessel pathology within 4.5 months since the initial diagnosis of a first cervical artery dissection, including recanalisation of an occlusion (44), progression of a stenosis to an occlusion (9) and dissection recurrence with new-onset occlusion or stenosis without pre-existing identical vessel pathology (9). Ten of those pathological changes were detected on the day of or the day after the first dissection, 19 more within the next 2 weeks. Another 13 had occurred by 6 weeks, and another 15 by 3 months after the first dissection (Figure 4).

**Figure 4:**
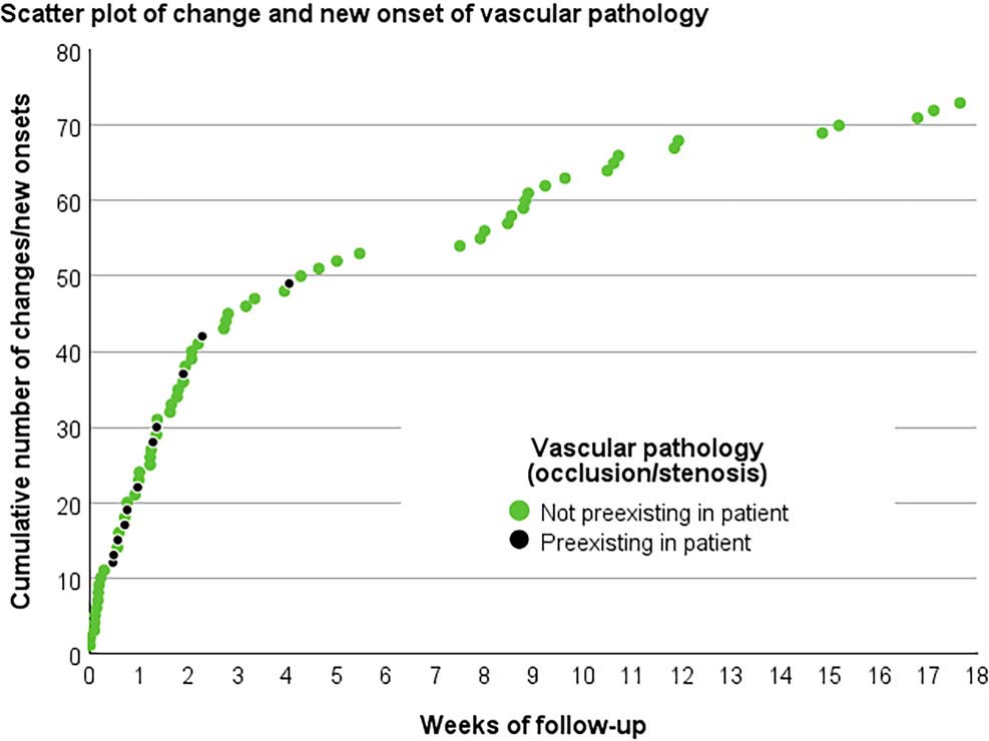
Scatter plot of change and new onset of vascular pathology (occlusion, stenosis) and time of their occurrence in 159 patients with spontaneous first cervical artery dissection.

## Discussion

In our study, a change from dissection-related occlusion to stenosis or vice versa, as well as the new onset of one of these 2 vascular pathologies as a result of recurrent dissection after an initial cervical artery dissection, was a common finding. This occurred in 18.2% of patients within 14 days and 26.4% within 6 weeks of the initial dissection. Around 60% of these changes occurred in the artery initially affected, while around 40% were due to recurrent dissections. In light of recent publications, this change in dissection-related vascular pathology over time—from occlusion to stenosis and vice versa—might gain therapeutic significance. A large observational study recently suggested fewer cases of ischaemia occurring under anticoagulation than under antiplatelet therapy in the presence of dissection-related occlusion which was not the case for dissection-related stenosis.^10^ This is remarkable given that 2 randomised studies comparing these 2 types of medication failed to demonstrate an advantage for either approach.^8^^,^^9^ In both studies, the existing pathology was not taken into account. Our results suggest that even stratifying therapy based on initial findings may not be sufficient. Frequent examinations, eg, every 2 days for the first 14 days and then weekly for up to 6 weeks after the initial dissection, may be necessary in order to adjust the medication to a rapidly changing vascular pathology within the first weeks after a cervical artery dissection.

Our study also shows that both causes of pathology change—recanalisation and recurrence—occur more frequently and at an earlier stage following the initial dissection than is reported in numerous observational studies on this topic. Regarding the spontaneous recanalisation of an occluded cervical artery, detected in 70.4% in our series, this is likely to be attributable to the very early start of the follow-up after initial dissection onset and the high frequency of examinations in the first weeks and months in our series.^11–14^ Our observed cumulative rate of recurrent dissection of 14.7% within 6 weeks is similar to other observations with prospectively planned frequent follow-up examinations in the first days and weeks after the initial dissection, ranging from 9.2% to 26.3%,^12^^,^^21^^,^^22^ but is remarkably higher than the rates reported in many studies without follow-up examinations starting promptly after the initial dissection and not performed with high frequency, ranging between 0.9% and 2.0%.^15^^,^^19^^,^^23^^,^^24^

Our data suggest that there is no association between recanalisation of dissection-related occlusion and administered anti-clotting medication, ie, intravenous thrombolysis, anticoagulants, and antiplatelet agents, in contrast to recanalisation of atherosclerotic ICA occlusion and antiplatelet agents.^25^ We also found no association between the progression of dissection-related stenosis to occlusion and intravenous thrombolysis, which makes an unwanted thrombolytic effect on vessel wall haematoma growth appear unlikely.

The cumulative rate of late recurrent dissection was 13.7% within a 10-year observation period, which is consistent with other long-term studies.^22^^,^^26^ Recurrences occurring within the first 6 weeks after the index event may be considered early recurrences, whereas those occurring thereafter may be regarded as late recurrences. The temporal separation between early and late recurrence of dissection in our cohort supports the hypothesis that early and late recurrence may represent distinct conditions.^22^^,^^27^ The appearance of an sCAD may indicate currently vulnerable vessel walls, increasing the likelihood of recurrence over several weeks. By contrast, late recurrent dissection may suggest an underlying vascular pathology specific to the patient. The different arteries affected by recurrent dissection also support this hypothesis. Early recurrences affected the corresponding contralateral artery in 85.0% of patients, whereas this pattern was observed in only 28.6% of late recurrences. This latter percentage can be explained by chance with respect to the 4 extracranial cervical arteries.

Recanalisation within the first 4 months and early recurrent dissection had no effect on the functional deficit after 4.5 months compared to the admission deficit. This could be due to the generally mild severity of the patients’ functional deficits in cases of dissection-related cerebral ischaemia, the significantly younger age of patients compared to those with other stroke aetiologies leading to a greater effect of rehabilitation measures on the functional outcome compared to other parameters, as well as an outcome scale that is not sufficiently sensitive to minor changes. Thus, early recanalisation up to hospital discharge in contrast to persisting occlusion was associated with significantly greater improvement of the neurological deficit.

Our study has limitations. It spans more than 2 decades, during which time the frequency of imaging modalities used to detect dissection changed substantially. Although we found no association between recanalisation/recurrence and the year of onset of dissection, this does not rule out the possibility of diagnostic bias. Patients were not systematically questioned or examined regarding migraine, fibromuscular dysplasia, other connective tissue disorders, a family history of cervical artery dissections or genetic markers. Numerous studies have shown associations between these factors and the occurrence of dissection.^12^^,^^15^^,^^27^^,^^28^ Furthermore, we have no data on the presence and extent of tortuosity of the cervical arteries in our patients, which has also been described as a predictor of recurrent dissections.^29^ Finally, the presence or absence of dissecting aneurysm was not systematically assessed. Thus, our findings on the temporal dynamic of pathology changes must be verified with respect to those variables. Even though both outcome parameters, recanalisation and recurrence, showed no time trend within the observation period, the lack of association with used antithrombotic drugs in our study should be confirmed by data with randomised drug administration. Treatment indication, eligibility and clinical practice for IVT evolved over the long inclusion period, which may have introduced heterogeneity related to treatment in analyses of clinical outcomes and vascular evolution. While we found no link between the year of dissection onset and the vascular outcomes examined, an impact of changes in acute treatment strategies over time may not be ruled out. We did not record whether spontaneous recanalisation or delayed occlusion was accompanied by clinical symptoms at the time it was detected. Whether a given change in vascular pathology was clinically silent or symptomatic can therefore not be determined from our data. Related to this, the NIHSS at discharge and the mRS at 4.5 months are indirect measures of the clinical relevance of recanalisation or occlusion: transient ischaemic attacks and clinically silent DWI-positive lesions are not necessarily reflected in either score, so that our clinical outcome measures are likely to underestimate the number of ischaemic events associated with changes in vascular pathology.

## Conclusion

Changing vessel pathology—from occlusion to stenosis and vice versa—and new onset pathologies—stenosis and occlusion—are frequent findings early after an sCAD. These findings support early vascular follow-up and provide a rationale for prospective studies investigating whether evolving vessel morphology should inform future antithrombotic strategies.

## Supplementary Material

Supplementary_material_aakag117

## Data Availability

The datasets generated and/or analysed during this study are not publicly available due to patient privacy and national data protection laws but are available from the corresponding author upon reasonable request.
