# Supplementary material for: Early change and new onset of vascular pathology in cervical artery dissection: implications for surveillance
Source: Eur Stroke J. 2026 Oct 5;11(10):aakag117. doi: 10.1093/esj/aakag117 (PMC13636090; doi:10.1093/esj/aakag117)
Supplement: Supplementary file 1 [file supplementary_material_aakag117.zip › Supplemental Material II_Table_ESJ_Revision-2_clean-copy.docx]

**Supplemental Material II - Table**

**Supplemental Table S1.** Therapy and clinical outcome in 159 consecutive patients admitted with first-time spontaneous cervical artery dissection

|  | N (%) |
| --- | --- |
| Intravenous thrombolysis | 30 (18.9) |
| Endovascular intervention | 29 (18.2) |
| Antithrombotic prevention (after dissection and during follow up) |  |
| Antiplatelet agents | 125 (78.6) |
| Anticoagulants | 10 (6.3] |
| Antiplatelet agents and anticoagulants (consecutively) | 23 (14.5) |
| None | 1 (0.6) |
| NIHSS score at discharge, Md (IQR) | 0 (0-4) |
| mRS at 4.5 months, Md (IQR) | 0 (0-2) |

IQR indicates interquartile range; MD, median; mRS, modified Rankin Scale; NIHSS, [American] National Institutes of Health Stroke Scale.
