# Supplementary material for: Early change and new onset of vascular pathology in cervical artery dissection: implications for surveillance
Source: Eur Stroke J. 2026 Oct 5;11(10):aakag117. doi: 10.1093/esj/aakag117 (PMC13636090; doi:10.1093/esj/aakag117)

Supplemental Material I - Figures

Supplemental Figure S1. Kaplan-Meier curves for time to spontaneous recanalization of acute dissection-related internal carotid artery (49 arteries) and vertebral artery occlusion (31 arteries).

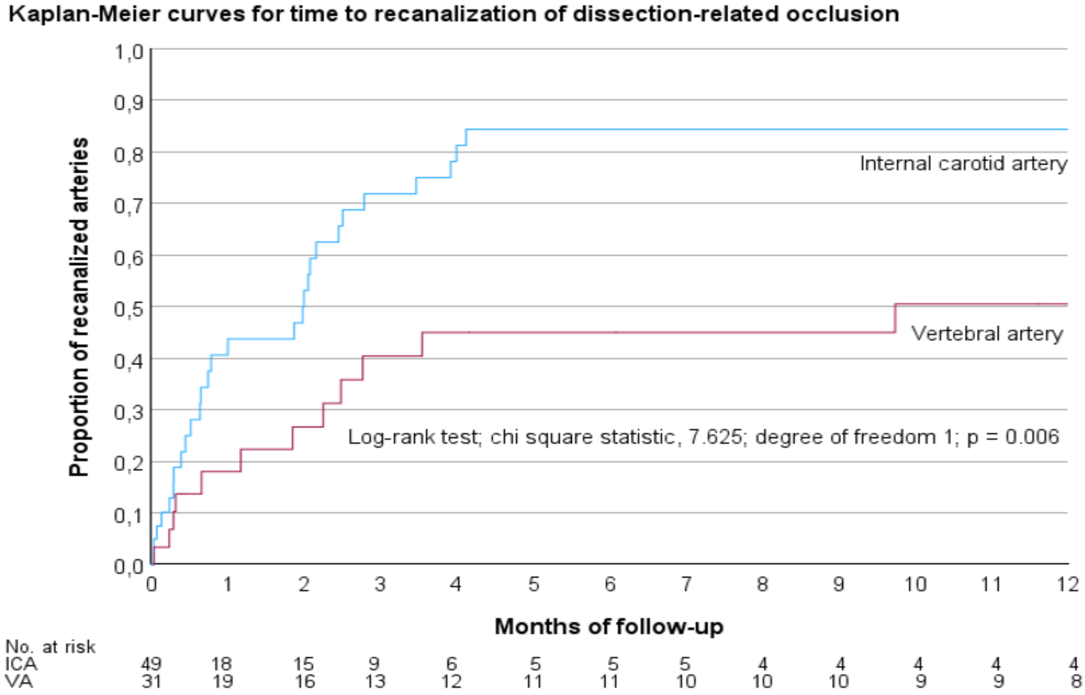

Supplemental Figure S2. Kaplan-Meier curves for time to spontaneous normalization of acute dissection-related cervical artery stenosis (109 arteries) and occlusion (80 arteries).

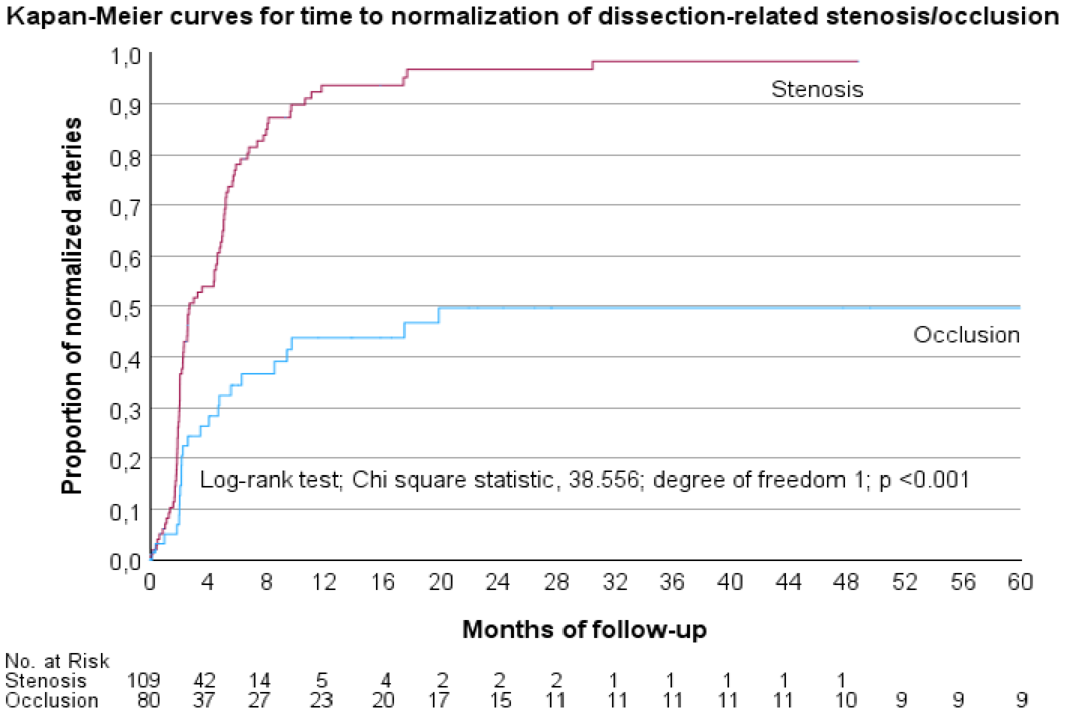

Supplemental Figure S3. Flow chart of the vascular course of 191 dissected cervical arteries.

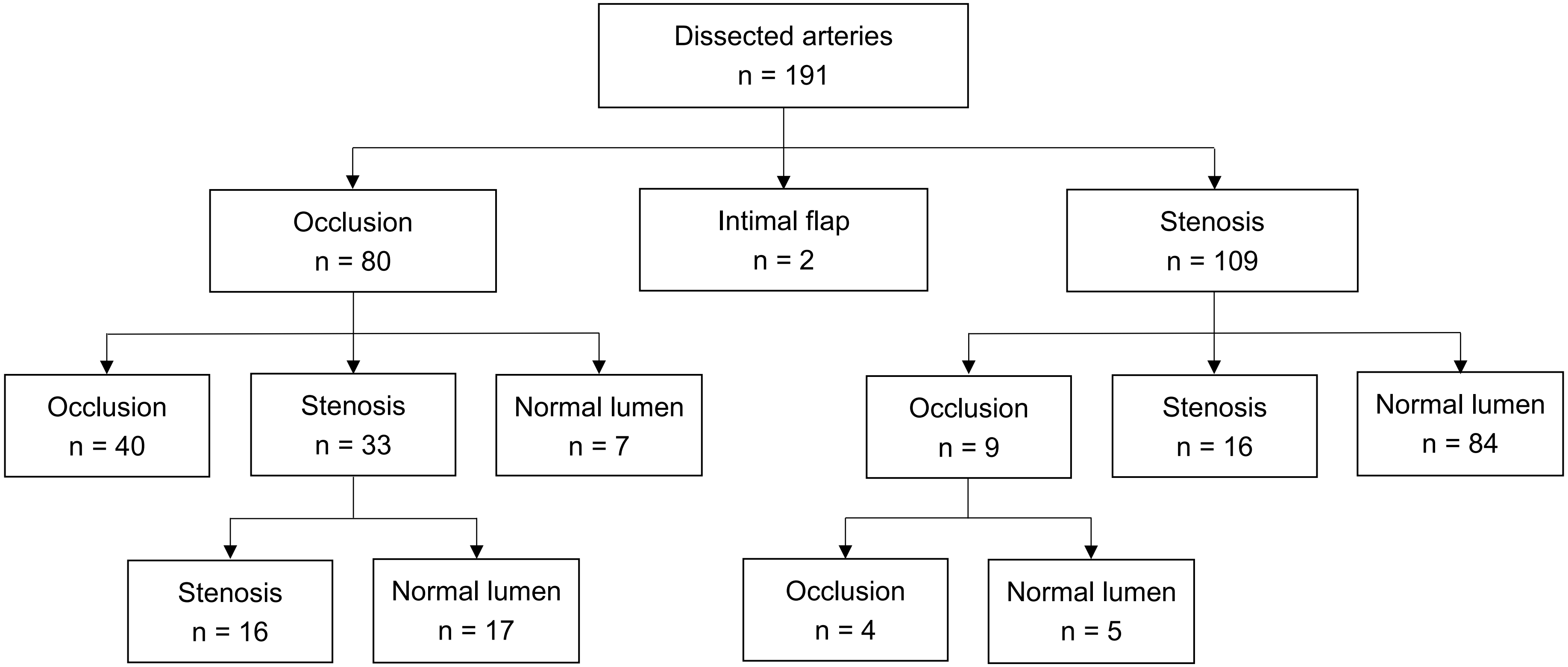

Supplemental Figure S4. Kaplan-Meier curve for time to first recurrence of cervical artery dissection in 159 patients.

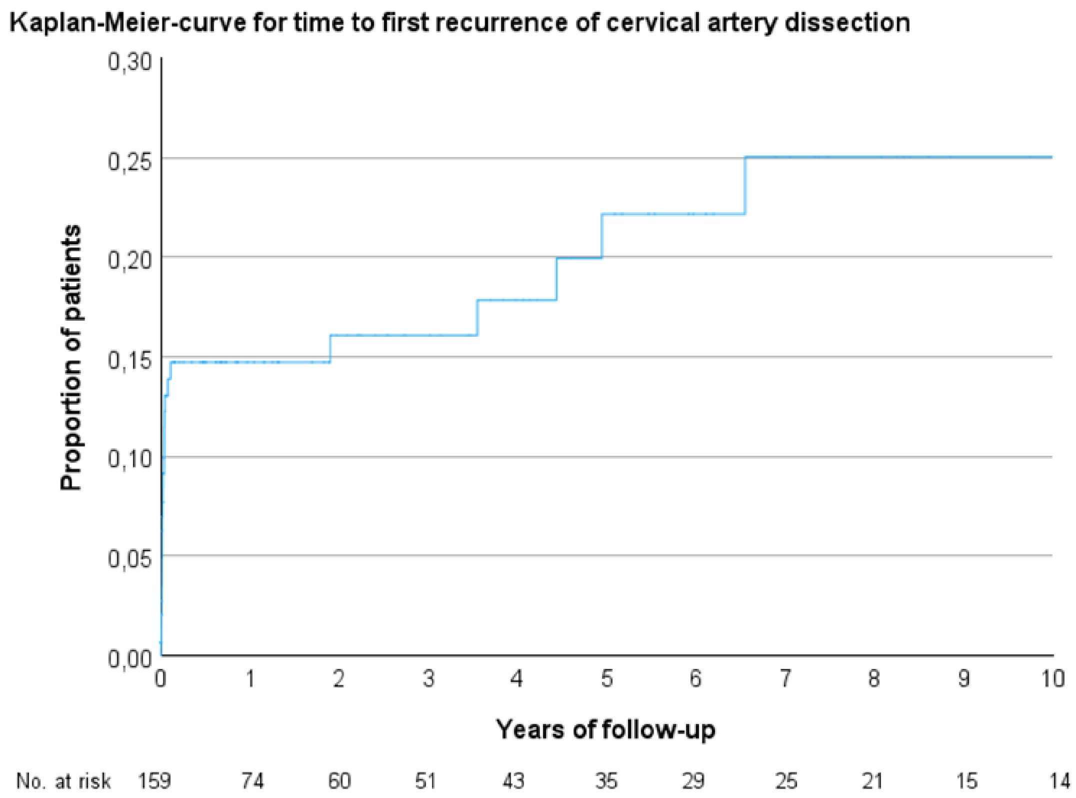

Supplement: Supplementary file 1 [file supplementary_material_aakag117.zip › Supplemental Material I_Figures.pdf]
